# The magic of love: gender differences in marriage duration and body mass index

**DOI:** 10.3389/fnut.2026.1867171

**Published:** 2026-10-19

**Authors:** Yuying Li, Panpan Li, Kaichao Shao

**Affiliations:** 1 Institute of Food and Strategic Reserves, Nanjing University of Finance and Economics, Nanjing, China; 2 School of Finance and Accounting, Yellow River Conservancy Technical University, Kaifeng, Henan, China

**Keywords:** BMI, gender difference, marital satisfaction, marriage duration, obesity

## Abstract

**Background:**

The global prevalence of obesity is rising, particularly among married individuals. However, limited research has explored the dynamic relationship between marriage and body weight, and the role of marital satisfaction in this relationship.

**Methods:**

Using 29,734 observations from the China Family Panel Studies (CFPS), this study examined the association between marriage duration (MD) and body mass index (BMI), as well as the moderating role of marital satisfaction. The analysis was guided by the health capital model and the Double ABC-X framework.

**Results:**

We found an inverted U-shaped relationship between MD and BMI, with a stronger association in men than in women. The association was more robust among couples with children and more pronounced in rural areas and northern China. Marital satisfaction buffered the association between MD and BMI in men, but not in women.

**Conclusion:**

MD has a nonlinear association with BMI, and this association differs by gender, household composition, and region. Marital satisfaction may serve as a protective factor for men's weight-related health. These findings inform the development of marriage-based weight management strategies.

## 1 Introduction

At present, obesity has become one of the most critical threats to public health (1). According to a report by the World Health Organization (2), in 2022, 1 in 8 people in the world were living with obesity (BMI ≥ 30 kg/m^2^). The trend is especially pronounced in China, where obesity is projected to increase at an annual rate of 5.4% from 2020 to 2035, ultimately reaching an adult obesity rate of 18% by 2035. The economic impact of overweight (BMI ≥ 25 kg/m^2^) individuals on China's GDP is estimated to be as high as $1.27 trillion, accounting for 3.1% of the total GDP (3). Given the pressing nature of this challenge, there is an increased urgency to investigate the social determinants of obesity.

From the perspective of family systems theory, the family operates as a holistic system of interconnected individuals whose behavior and health are deeply embedded in family interactions (4). Marriage, as the core dyadic relationship within the family, may influence spouses' weight status through shared lifestyles, socioeconomic resources, and marital stress (5–9). Numerous studies have shown that marital status, marital satisfaction, and the quality of communication between couples are strongly associated with changes in the weight of both men and women (10–15). As obesity becomes increasingly prevalent in China, the relationship between marriage and body weight has attracted significant research attention. Prior research has documented a positive association between marriage and overweight/obesity in China, with stronger effects consistently observed among men than among women (14, 16). However, most of these studies have relied on static binary indicators of marital status, thereby overlooking the evolving nature of the marriage–weight relationship across marital duration and the potential moderating role of marital quality.

This study uses unbalanced panel data from the China Family Panel Studies (CFPS), utilizing 29,734 observations from the 2014, 2018, 2020, and 2022 survey waves. Building upon this data, we contribute to the existing literature by examining the relationship between marriage duration and BMI in China, its underlying heterogeneity, and the moderating role of marital satisfaction. The contributions of this study are 3-fold. First, we integrate Grossman's model of the demand for health with the Double ABC-X model of family stress framework to theorize an inverted *U*-shaped relationship between marriage duration and BMI, offering a plausible explanation for the dynamic relationship between marriage and body weight (17, 18). Second, drawing on dyadic coping theory, we incorporate marital satisfaction as a moderator to test whether relationship quality buffers or amplifies the association between marriage duration and BMI, thereby offering evidence on the conditions under which marriage promotes or mitigates weight gain. Third, we examine gender and regional differences in the association between marriage duration and BMI using CFPS data, identifying contextual and demographic boundary conditions of the marital health effect.

## 2 Theoretical framework

### 2.1 Marriage and weight

Marital relationships are strongly associated with health outcomes across the life course and have cumulative effects on health over time (19). McCubbin and Patterson's (18) Double ABC-X model of family stress process emphasizes the protective function of marriage, whereby the family will buffer external stressors (aA) by mobilizing resources (bB) and reconfiguring cognition (cC) to maintain overall physical and mental health. Grossman (17) proposed the theory of the demand for health—commonly referred to as the health capital model, which views health as a form of capital that individuals can accumulate through deliberate investments. Therefore, the impact of marriage on health investment varies based on the intertemporal trade-offs that families navigate within their resource limitations. Drawing on previous research, we integrate the theory of the demand for health with the Double ABC-X model of family stress process to elucidate the phenomenon of weight change within marriages (see Figure 1). This framework posits that individuals engage in health capital investment decisions within the family unit, a process influenced by the family's perceptions, resource allocation, and cognitive adaptation to ongoing stress. We draw on this integrated framework primarily as a heuristic device to guide the interpretation of our empirical findings, while recognizing that direct measures of its core constructs (e.g., stressors, coping resources, and cognitive appraisals) are not available in our data.

**Figure 1:**
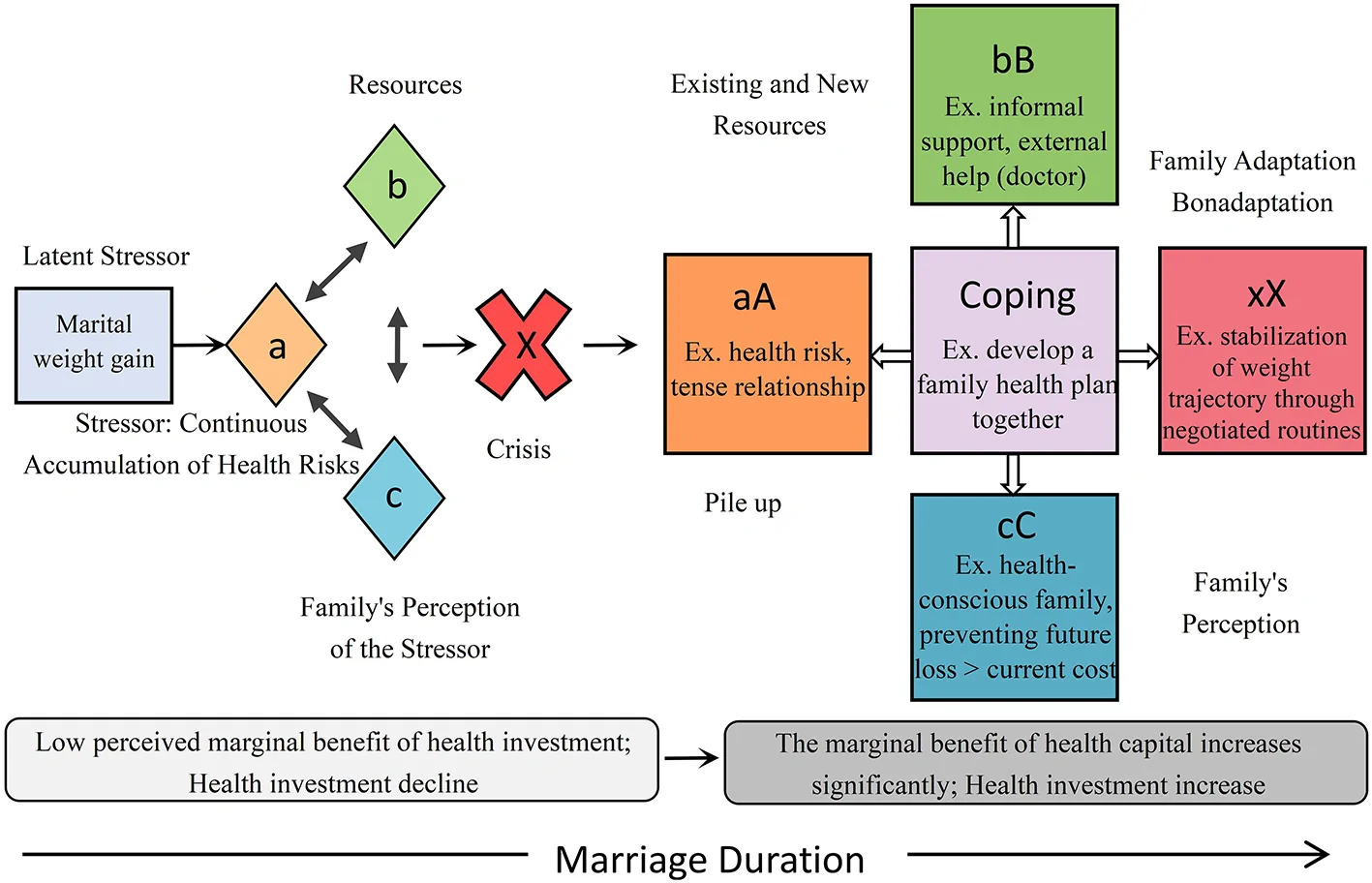
The dynamic trajectory of marital weight change. This figure integrates the Double ABC-X model of family stress and adaptation with Grossman”s health capital theory to depict the dynamic trajectory of marital weight change. Initially, weight gain functions as a latent stressor—unrecognized and unaddressed due to low perceived marginal benefits of health investment relative to its opportunity costs (e.g., time, effort). As marriage duration increases, cumulative health risks and relational strains eventually cross a threshold, activating the stressor (a′) and triggering the ABC-X crisis process. Over time, the marginal benefit of maintaining health capital rises (e.g., avoiding future disability, preserving caregiving capacity), prompting families to reconfigure resources (bB) and cognitively reframe health as a shared asset (cC). Successful adaptation (xX)—such as establishing joint routines that stabilize weight—reflects Bonadaptation. Source: Adapted from Ballard et al. (76), licensed under CC BY-NC-SA 4.0 (https://creativecommons.org/licenses/by-nc-sa/4.0/), and based on McCubbin and Patterson (18).

Individuals typically enter marriage with a relatively lower BMI (20, 21). However, in the early stages of marriage, the dual pressures of family building and personal development drive up the opportunity cost of health capital investment. At the same time, slow weight gain has not yet caused perceptible functional impairment or relationship conflict, and its health depreciation effects are underestimated, resulting in lower expected marginal benefits of healthy behaviors. Family resource allocation prioritizes market labor and childcare activities, and health investments are squeezed out, which is manifested in a decrease in healthy food consumption, a significant increase in high-calorie foods, and a decrease in the frequency of physical exercise activities (22–24). During this phase, the rate of health capital accumulation decelerates, or even net depreciation accelerates, with one outward manifestation being an increase in body fat percentage or weight gain (23, 25–27).

It is worth noting that stress and weight may reinforce each other through a bidirectional feedback loop. The accumulated burden of excess weight itself can serve as a chronic stressor. Empirical studies have shown a U-shaped non-linear relationship between BMI and perceived depression. And overweight or obesity significantly increases household medical expenditures as well as the risk of catastrophic health expenditure (28–30). This suggests that BMI above the healthy threshold may act as a family stressor (a), for example through increased medical expenses and psychological strain. At this point, if the family can mobilize sufficient resources (b) and form a consistent risk perception (c), it may prevent further deterioration through adaptive adjustments. Resources may include time, financial support, and health management capacity. Risk perception refers to shared awareness of health consequences, such as the increased risk of diabetes and cardiovascular disease associated with being overweight.

Conversely, resource constraints (b) and cognitive divergence (c) may trigger a short-term crisis (X). For example, resources may be constrained when couples allocate time and money to childcare or caring for elderly parents, and cognitive divergence may manifest as conflicts in health priorities. As stress persists without relief, weight-related burdens may accumulate into long-term chronic stress (aA). This stress not only manifests as potential risks in medical expenditures but may also be associated with declining marital satisfaction (31, 32), together contributing to an overall stress load (aA). At this point, the potential value of averting future medical costs and relational deterioration becomes salient, as discounted health-loss costs surpass lifestyle adjustment costs, raising the expected marginal return on health investment. This provides households with stronger incentives to accumulate health capital, consistent with rational intertemporal decision-making. In this context, as marriage continues, families gradually develop adaptive coping strategies. On the one hand, families may implement substantive behavioral interventions through resource reallocation (bB). Examples include developing joint family health plans that encourage couples to coordinate dietary adjustments and increase regular exercise (33). Families may also provide support for bariatric surgery and postoperative management when severe obesity poses health crises (34, 35). On the other hand, families engage in cognitive restructuring (cC), redefining weight management as the maintenance of common family assets, a process that may translate perceived health threats into weight management behavioral intentions (36). These pathways may be associated with the stabilization or reduction of weight in later stages of marriage, although further research is needed to validate these mechanisms.

Existing studies have used a single variable of whether they are married or not, ignoring the dynamic change process of the long-term social relationship of marriage on body weight, which can easily lead to sample selection bias and causal identification bias. For instance, the effect of marriage on weight may vary considerably across life stages, with distinct mechanisms at play during the honeymoon period and during the transition to parenthood (23, 25, 37). Such simple binary classification cannot capture this within-group heterogeneity, which may lead to bias in the estimated average treatment effects. To address this, we include marriage duration and its non-linear terms to capture the dynamic trajectory of marital influences on body weight. It is worth noting that weight changes among couples often exhibit synchronized fluctuations due to shared environments (38). However, the underlying mechanisms are typically driven by individuals' subjective perceptions of and responses to stress and health risks. Therefore, this study focuses on analyzing the relationship between marriage duration and body weight at the individual level. Based on the above analysis, the following hypotheses are put forward:

Hypothesis 1: There is a non-linear, inverted *U*-shaped association between marriage duration and BMI.

### 2.2 Gender differences in weight within marriage

“There are two marriages, then, in every marital union, his and hers (39).” Gender role theory points out that society endows men and women with different family roles and behavioral expectations through cultural norms and institutional arrangements. In intimate relationships, men often take on the role of the family breadwinner, with their value being predominantly associated with income, status, and contributions to the family economy rather than body shape maintenance (40). Consequently, post-marriage, men may pay less attention to weight management, and this reduced focus is associated with weight gain (16).

The relationship between marriage and weight outcomes is more intricate for women than for men, shaped by countervailing forces. On the protective side, marriage provides economic support and emotional value (41, 42). And women in marriage often act as family health gatekeepers, potentially reinforcing healthy behaviors (33, 40). On the damaging side, the dual burden of paid work and unpaid domestic labor exacerbates time scarcity and chronic stress for women, crowding out their investments in exercise and nutrition (43–46). Sociocultural norms add another layer of complexity. Women are often pressured to stay thin because society closely links their appearance to social value (47, 48). Moreover, studies show that overweight women face greater penalties than men, including discrimination in employment and marriage (49–51).

In summary, the logic underlying post-marital weight changes is predominantly shaped by the breadwinner role for men, whereas women are subject to the dual constraints of domestic caregiving responsibilities and sociocultural norms of thinness. That is, the pathways through which marriage affects body weight differ significantly between men and women. For instance, using Chinese data, Quan and Zhang (16) document that marriage is significantly associated with obesity risk only for men. This pattern is consistent with Sobal et al. (52) for the U.S. Accordingly, the following hypothesis is proposed:

Hypothesis 2: There is gender heterogeneity in the relationship between marriage duration and BMI.

### 2.3 A satisfied marriage and weight

Marriage involves deep emotional connections, and its quality is significantly associated with individual health outcomes. The dyadic coping theory emphasizes collaborative engagement between partners in joint stress management. Satisfied couples show more positive support-oriented dyadic coping during stressful events, whereas distressed couples predominantly exhibit negative dyadic coping behaviors (53, 54). Existing literature indicates that higher-quality marriages are associated with better health outcomes (55, 56).

For weight, the influence of marital satisfaction runs through the entire marriage. In the early stage of marriage, when weight has not yet become an explicit stressor (a), highly satisfying marriages are often accompanied by increased life stability and enhanced emotional sharing, which may be associated with a certain degree of weight gain. Concurrently, high-quality intimate relationships are linked to a range of favorable physiological and psychological characteristics, including lower perceived stress levels, better sleep quality, improved emotional wellbeing, and reduced cortisol secretion along with a decreased risk of related metabolic disorders (57–59).

When weight becomes a source of stress for an individual, manifesting as body anxiety or health issues, marital satisfaction may indirectly influence individual health behaviors and weight changes by moderating the interaction patterns between spouses. Specifically, when weight accumulation develops into individual stress (body image anxiety, health problems, etc.), couples with high marital satisfaction tend to be actively involved, preferring collaborative coping strategies, effective deployment of supportive resources (bB), and constructing positive cognitive conceptions of stress (cC) (11, 12). Prior research has documented that spousal emotional support is correlated with reduced negative perceptions of one's own body shape and is also positively associated with partners' sustained motivation for weight loss (12). Meltzer et al. (23), in their 4-year longitudinal study of 169 newlywed couples, found that the spouse's positive supportive behavior was significantly related to slower weight gain, highlighting the protective effect of high-quality marriage. By contrast, low-quality marriages exhibits a positive correlation with multiple health risk indicators, such as negative thought patterns, psychological stress, sleep disorders, and emotional eating (10, 60, 61). Kouvonen et al. (56) found that participants with high levels of negative intimate relationships were more likely to experience the transition from overweight to obesity. Therefore, marital satisfaction does not merely reflect relationship status, but is a key moderator in shaping a healthy behavioral environment. Based on this, we propose the following hypothesis:

Hypothesis 3: Marital satisfaction moderates the relationship between marriage duration and BMI, and this moderating effect is gender-specific.

## 3 Data and methods

### 3.1 Data

The data in this paper are from the CFPS. The CFPS Baseline Survey was officially implemented nationwide in 2010. It is a nationwide and comprehensive social tracking survey project with random samples drawn from 25 provinces/municipalities/autonomous regions that account for 95% of China's total population. All family members identified in the baseline survey, along with their future biological or adopted children, are enrolled as CFPS panel members and are longitudinally tracked through biennial surveys. The tracking and collection of data at the individual, family, and community levels reflect the changes in China's society, economy, demographics, education, and health.

The CFPS has now released seven full rounds of survey data for the 2010, 2012, 2014, 2016, 2018, 2020, and 2022 surveys. First, considering the purpose of the study in this paper, the years that lacked the necessary explanatory variables were excluded. The marital satisfaction variable in this study is based on the question “Overall, how satisfied are you with your current married life?” This item was not included in 2010, 2012, and 2016. Therefore, in order to maintain the consistency of the data, we finally chose the data of 2014, 2018, 2020, and 2022. Secondly, to account for the potential influence of age on metabolic rate, we restricted our sample to married adults aged 20 to 60, as metabolic levels remain relatively stable within this age range (62). In addition, we excluded samples with missing height, weight, and date of marriage. To reduce the influence of extreme values, continuous variables were truncated by trimming 1% of observations at both tails. Finally, to avoid confounding from marital status transitions, we excluded individuals who had divorced or remarried during the study period. The final analytic sample comprised 10,716 married individuals who were interviewed at least twice across the four survey waves, yielding a total of 29,734 observations.

### 3.2 Variables

#### 3.2.1 Explained variable

Weight. Three indicators were used to assess weight status. First, use the BMI (Body Mass Index), which is calculated by dividing a person's weight (in kilograms) by the square of their height (in meters). Second, use the overweight/obesity status, represented by a binary categorical variable. According to the classification standards of BMI characteristics for the Chinese population by the Chinese Nutrition Society, individuals with a BMI below 24 kg/m^2^ are assigned a value of 0, and individuals with a BMI greater than or equal to 24 are assigned a value of 1, indicating overweight/obesity. Third, we also used body weight (in kg) as an alternative measure to further validate our findings.

#### 3.2.2 Explanatory variable

Marriage duration (MD). In this article, we use marital duration (MD) to evaluate the respondents' marital life. The marital duration is calculated based on the marriage date, with the year of marriage counted as the initial year (MD = 1).

#### 3.2.3 Adjustment variables

Marital satisfaction (MS). In this study, marital satisfaction was assessed using the survey question: “How satisfied are you with your current marital life?” Responses were recorded on a 5-point Likert scale ranging from 1 (very dissatisfied) to 5 (very satisfied).

#### 3.2.4 Control variables

Individual level: including gender, age, education, health level, smoking, and alcohol consumption. Details are as follows:

Education: derived from self-reported highest educational attainment, converted to total years based on China's standard educational system (e.g., 6 years for primary school, 3 years for junior high).

Health: measured via self-reported health status, categorized into five ordered levels: 1 (“Poor”), 2 (“Fair”), 3 (“Good”), 4 (“Very good”), and 5 (“Excellent”).

Smoking: A binary variable derived from the question, “*Have you smoked any tobacco products in the past 30 days?”* Responses were coded as 1 (“Yes”) or 0 (“No”).

Drinking: a binary variable derived from the question, “*In the past month, did you drink alcohol more than 3 times a week?”* Responses were coded as 1 (“Yes”) or 0 (“No”).

Household level: including family size, household income, Engel coefficient, and the number of children.

Household income: measured as net household income, log-transformed after adding 1.

Engel Coefficient: defined as food expenditure divided by total household expenditure. A lower Engel coefficient generally indicates that a household has more resources available for other consumption, savings, or investment after meeting basic food needs, thus reflecting a higher standard of living and greater economic capacity.

### 3.3 Models

In the analysis, marital duration and BMI were treated as continuous variables and analyzed using Ordinary Least Squares (OLS) regression (Equation 1). To account for the potential non-linear effects of age on BMI, we include age-group fixed effects (for groups ≤ 30, 31–40, 41–50, and 51–60 years) in the model. The estimating equation is specified as follows:


$$\begin{array}{l} BMI_{it}=\alpha_{0}+\alpha_{1}MD_{it}+\alpha_{2}MD_{it}^{2}+\beta^{\prime }X_{it}+\lambda_{p}+\mu_{t} \end{array}\begin{array}{l} +\gamma_{g}+\varepsilon_{it} \end{array}$$ (1)

In the above model, *BMI*_*it*_ denotes the BMI of individual *i* at time *t*. MD_*it*_ represents the individual's duration of marriage, *X*_*it*_ is a vector of control variables, including both individual and household characteristics. α_0_, α_1_, α_2_, β are the parameters to be estimated. λ_*p*_ is the provincial fixed effect. μ_*t*_ is the year fixed effect. μ_*t*_ denotes the age-group fixed effects. ε_*it*_ is the random error term.

To examine the moderating effect of marital satisfaction, we extended the baseline model by adding interaction terms between marriage duration (and its squared term) and marital satisfaction. Following Aiken and West (63), we mean-centered both marriage duration and marital satisfaction before constructing the interaction terms to reduce multicollinearity and ease the interpretation of the lower-order coefficients.


$$BMI_{it}\;=\alpha_{0}+\alpha_{1}MD_{it}+\alpha_{2}MD_{it}^{2}+\alpha_{3}MS_{it}+\alpha_{4}MD_{it}\times MS_{it}+\alpha_{5}MD_{it}^{2}\times MS_{it}+\beta^{\prime }X_{it}+\lambda_{p}+\mu_{t}+\gamma_{g}+\varepsilon_{it}$$ (2)

In the Equation 2, *MD*_*it*_ and *MS*_*it*_have been mean-centered prior to the construction of the interaction terms.

### 3.4 Descriptive statistics

Table 1 reported descriptive statistics for each variable. This study employed an unbalanced panel dataset with a total of 29,734 observations. The mean BMI of the sample was 23.67, and the proportion of overweight/obese individuals was 43.49%. The average age of the respondents was 44.36 years. Among the married respondents, 48.75% were male and 51.25% were female. The mean marriage duration was 23.15 years.

**Table 1:** Sample characteristics: descriptive statistics of key variables.

Variable	Definition	Obs	Mean	SD	Min	Max
Marriage						
Marriage duration	Duration of current marriage (in years)	29,734	23.15	10.55	1	45
Weight status of full sample						
BMI	Weight/height^2^ (kg/m^2^)	29,734	23.67	3.29	10.82	45
Overweight/Obesity	0 = BMI < 24, 1 = BMI?24	29,734	0.43	0.50	0	1
Weight	Weight (kg)	29,734	64.30	11.32	27	120
**Instrumental variable**						
Relative age at first marriage	The absolute difference between the individual's actual age at first marriage and the provincial average age at first marriage in 2010	29,734	13.31	10.40	0.03	38.30
**Moderating variable**						
Marital satisfaction	Marital satisfaction in current life [from 1 (very dissatisfied) to 5 (very satisfied)]	29,734	4.44	0.92	0	5
**Individual level**						
Gender	0 = female, 1 = male	29,734	0.49	0.50	0	1
Age	Actual age (years old)	29,734	44.36	9.80	20	60
Education	Education background (years)	29,734	8.87	4.10	0	22
Health	State of health (1 = “Poor”, 2 = “Fair”, 3 = “Good”, 4 = “Very Good”, 5 = “Excellent”)	29,734	3.08	1.16	1	5
Smoking	Tobacco product use in the past 30 days (Yes = 1, No = 0)	29,734	0.29	0.45	0	1
Drinking	drink alcohol more than 3 times a week (Yes = 1, No = 0)	29,734	0.15	0.36	0	1
**Family level**						
Family size	Number of family members	29,734	4.41	1.91	1	16
Household income	Ln(Household Net Income + 1)	29,734	11.21	0.94	0	15.61
Engel Coefficient	Food expenditure (yuan/Year)/Total household expenditure (yuan/Year)	29,734	0.31	0.14	0	0.9
Number of children	The number of children raised by family	29,734	1.76	0.81	0	9
**Region**						
Urban	0 = rural, 1 = urban	29,734	0.50	0.50	0	1
South	0 = North, 1 = South	29,734	0.39	0.49	0	1

This table reports the sample definition, size (Obs.), mean, standard deviation (SD), minimum, and maximum for each variable. BMI, body mass index. The instrumental variable is defined as the difference between the individual's actual age at first marriage and the provincial average age at first marriage in 2010.

The data for each of the other variables were as follows: in terms of individual characteristics, the average age was 44.36 years; the average education level was 8.87 years; the average level of health was 3.08; 29% smoked cigarettes and 15% drank alcohol. Regarding family characteristics, the average household size was 4.41 persons; the average log-transformed household income [i.e., ln(income + 1)] was 11.21; the average Engel coefficient per household was 0.31; and the average number of children per household was 1.76. In addition, 39% of the population live in the South, and 50% live in urban areas.

## 4 Results

### 4.1 Baseline regression

Table 2 reports the baseline regression results. Across the full sample (Column 1), males (Column 2), and females (Column 3), the linear coefficients of marriage duration are positive, while the quadratic coefficients are negative, and all are statistically significant at the 1% level. The linear coefficients are 0.0638 (full sample), 0.0764 (men), and 0.0703 (women), with corresponding quadratic coefficients of −0.0013, −0.0016, and −0.0014, respectively (all *p* < 0.01). This finding indicates an inverted U-shaped relationship between marriage duration and BMI. And the linear effect of marriage duration on BMI exhibits a notable gender difference, with the estimated linear coefficient for men being approximately 8.68% higher than that for women, suggesting a stronger positive association between marriage duration and BMI among men.

**Table 2:** The relationship between marriage duration and BMI.

	**BMI**		
Variables	All	Men	Women
	(1)	(2)	(3)
Marriage duration (MD)	0.0638^***^	0.0764^***^	0.0703^***^
	(7.5247)	(6.3444)	(5.6118)
MD^2^	−0.0013^***^	−0.0016^***^	−0.0014^***^
	(−6.5295)	(−5.6290)	(−5.0065)
Gender	0.8798^***^		
	(13.1380)		
Age	0.0058	−0.0125	0.0209^*^
	(0.7826)	(−1.3096)	(1.8586)
Education	−0.0042	0.0532^***^	−0.0405^***^
	(−0.5541)	(4.8732)	(−3.8810)
Health	−0.0348^***^	−0.0086	−0.0545^***^
	(−2.8375)	(−0.4793)	(−3.2396)
Smoking	−0.1803^***^	−0.2002^***^	0.0501
	(−3.4552)	(−3.7473)	(0.2284)
Drinking	0.0901^**^	0.0962^**^	0.1686
	(2.2327)	(2.2320)	(1.4569)
Family size	−0.0260^***^	−0.0312^***^	−0.0198
	(−2.8526)	(−2.6166)	(−1.4093)
Household incomed	0.0427^**^	0.0869^***^	0.0044
	(2.4363)	(2.8302)	(0.2147)
Engel coefficient	−0.0022	0.1086	−0.0938
	(−0.0273)	(0.9370)	(−0.8391)
Number of children	−0.0656^**^	−0.0595	−0.0779^*^
	(−2.0180)	(−1.3518)	(−1.6484)
Year fixed effect	YES	YES	YES
Province fixed effect	YES	YES	YES
Age-group fixed effect	YES	YES	YES
*N*	29734	14497	15237
*R* ^2^	0.0743	0.0715	0.0884

Coefficients are reported with Z-statistics in parentheses. ^*^p < 0.10, ^**^p < 0.05, ^***^p < 0.01.

To further verify the statistical reliability of the inverted *U*-shaped relationship and to precisely locate the turning point, we employed the *U*-test proposed by Lind and Mehlum (64). The results show that the inverted *U*-shaped relationship is statistically significant at the 1% level across the full sample, men, and women, with estimated turning points of approximately 24.5, 23.9, and 25.1 years of marriage, respectively.

The subsample regressions reveal substantial gender heterogeneity in the association between marriage duration and body weight. While both men and women display an inverted *U*-shaped pattern, the linear coefficient of marriage duration is larger for men than for women, implying that the positive weight-related effect of marriage is stronger and more persistent among men. To formally test the statistical significance of this gender difference, we estimated a pooled model including an interaction term between sex and marriage duration. The coefficient on the interaction term was statistically significant at the 1% level, confirming that the linear effect of marriage duration on BMI differs significantly between men and women. These findings provide support for both Hypothesis 1 and Hypothesis 2.

### 4.2 Additional robustness checks

To ensure the reliability of the baseline regression results, we perform the following robustness checks in this paper.

**Substitution of variables method**. To ensure that our core findings are not driven by the choice of BMI as the primary outcome measure, we conducted robustness checks by replacing the dependent variable with two alternative indicators. Specifically, we re-estimated the baseline model using (1) body weight in kilograms (kg) as a continuous outcome, and (2) a binary indicator of overweight/obesity (overweight/obese = 1, normal weight = 0) as the dependent variable, estimated via a linear probability model.

Table 3 reports the robustness check results with alternative dependent variables, and the core findings remain robust. In the full sample, Columns 1 and 4 show that marriage duration exhibits a significant inverted *U*-shaped relationship with both body weight and the probability of overweight/obesity, consistent with the baseline results. Subsample analyses further confirm significant gender heterogeneity. Specifically, the coefficient on the linear term of marriage duration for body weight is 42.49% larger for males than for females, and that for the risk of overweight/obesity is 16.67% larger for males. Overall, these results corroborate our baseline results, with no substantive changes in the core conclusions even after alterations in the dependent variable.

**Table 3:** Robustness test: alternative dependent variable.

	**BMI**→**Weight**			**BMI**→**Overweight/Obesity**		
Variables	All	Men	Women	All	Men	Women
	(1)	(2)	(3)	(4)	(5)	(6)
Marriage duration (MD)	0.1869^***^	0.2485^***^	0.1744^***^	0.0070^***^	0.0091^***^	0.0078^***^
	(7.0634)	(6.3627)	(4.9834)	(4.9771)	(4.5314)	(4.0130)
MD^2^	−0.0037^***^	−0.0052^***^	−0.0034^***^	−0.0001^***^	−0.0002^***^	−0.0001^**^
	(−6.5441)	(−6.0139)	(−4.6004)	(−3.1103)	(−3.5324)	(−2.4751)
Gender	10.5686^***^			0.1202^***^		
	(51.3670)			(11.5489)		
Age	−0.0566^***^	−0.1386^***^	0.0161	−0.0013	−0.0025	−0.0002
	(−2.6400)	(−4.5219)	(0.5564)	(−1.1209)	(−1.5461)	(−0.1056)
Education	0.0923^***^	0.2946^***^	−0.0238	−0.0005	0.0101^***^	−0.0069^***^
	(4.1492)	(8.3763)	(−0.8528)	(−0.4035)	(5.8249)	(−4.6529)
Health	−0.0395	0.0302	−0.0902^**^	−0.0063^***^	−0.0013	−0.0093^***^
	(−1.2406)	(0.6132)	(−2.1964)	(−2.6843)	(−0.3784)	(−2.8711)
Smoking	−0.3148^**^	−0.3792^**^	0.6932	−0.0325^***^	−0.0330^***^	−0.0086
	(−2.0125)	(−2.3630)	(1.0880)	(−3.3188)	(−3.1996)	(−0.2684)
Drinking	0.2517^**^	0.2420^*^	0.5739^**^	0.0043	0.0113	−0.0115
	(2.1679)	(1.9015)	(2.1820)	(0.5738)	(1.3819)	(−0.5919)
Family size	−0.0801^***^	−0.1024^***^	−0.0551	−0.0038^**^	−0.0064^***^	−0.0007
	(−3.2569)	(−2.8830)	(−1.6434)	(−2.2098)	(−2.6486)	(−0.2966)
Household income	0.1791^***^	0.3259^***^	0.0509	0.0057^*^	0.0127^***^	−0.0015
	(3.7721)	(3.5725)	(1.0694)	(1.9367)	(2.7152)	(−0.4116)
Engel coefficient	0.1829	0.3394	0.0677	0.0040	0.0142	−0.0026
	(0.8771)	(1.0382)	(0.2606)	(0.2496)	(0.6168)	(−0.1200)
Number of children	−0.1315	−0.1408	−0.1436	−0.0044	−0.0054	−0.0047
	(−1.4202)	(−1.0450)	(−1.1446)	(−0.8277)	(−0.7066)	(−0.6322)
Year fixed effect	YES	YES	YES	YES	YES	YES
Province fixed effect	YES	YES	YES	YES	YES	YES
Age-group fixed effect	YES	YES	YES	YES	YES	YES
*N*	29,734	14,497	15,237	29,734	14,497	15,237
*R* ^2^	0.2939	0.1413	0.0968	0.0564	0.0613	0.0707

Coefficients are reported with Z-statistics in parentheses. ^*^p < 0.10, ^**^p < 0.05, ^***^p < 0.01.

**Exclusion of specific subsamples**. To further rule out potential confounding from sample selection bias and external macroeconomic shocks, we conducted two robustness checks by excluding specific subsamples. First, we excluded municipalities and autonomous regions. Given that municipalities differ substantially from other provinces in terms of administrative status and economic scale, and that autonomous regions exhibit distinct policy environments and demographic compositions, we excluded Shanghai, Beijing, Tianjin, Chongqing, Inner Mongolia, and Guangxi to enhance sample homogeneity. Second, we excluded the 2020 and 2022 survey waves (the COVID-19 pandemic period) to eliminate potential disruptions to weight-related behaviors and health outcomes caused by the public health crisis.

Table 4 reports the robustness check results after excluding specific subsamples, and the core findings remain robust. In the full sample, Columns 1 and 4 show that after excluding municipalities/autonomous regions and the COVID-19 period samples, the inverted U-shaped relationship between marriage duration and BMI remains significant, confirming the reliability of the baseline results. Specifically, in the sample excluding municipalities/autonomous regions, the linear coefficient of marriage duration for BMI is 11.01% larger for males (Column 3) than for females (Column 2). In the 2020–2022 excluded sample, this gender gap increases to 25.20% (Columns 5 and 6). These results again demonstrate that our findings are robust to alternative sample compositions and the exclusion of observations during the COVID-19 pandemic.

**Table 4:** Robustness test: exclusion of specific subsamples.

	BMI (Excluding certain provinces)			BMI (Excluding 2020 and 2022)		
Variables	All	Men	Women	All	Men	Women
	(1)	(2)	(3)	(4)	(5)	(6)
Marriage duration (MD)	0.0689^***^	0.0817^***^	0.0736^***^	0.0695^***^	0.0939^***^	0.0750^***^
	(7.4659)	(5.9947)	(5.9274)	(5.8992)	(5.3512)	(4.6823)
MD^2^	−0.0013^***^	−0.0016^***^	−0.0014^***^	−0.0011^***^	−0.0016^***^	−0.0012^***^
	(−6.3851)	(−5.1685)	(−5.3028)	(−3.5237)	(−3.7951)	(−3.1001)
Gender	0.8430^***^			0.9736^***^		
	(12.0715)			(12.1803)		
Age	0.0031	−0.0127	0.0160	0.0023	−0.0076	0.0125
	(0.4028)	(−1.2732)	(1.3561)	(0.2333)	(−0.5642)	(0.8727)
Education	−0.0056	0.0494^***^	−0.0388^***^	−0.0044	0.0486^***^	−0.0340^***^
	(−0.7168)	(4.3539)	(−3.5609)	(−0.5100)	(3.8313)	(−2.8814)
Health	−0.0348^***^	−0.0073	−0.0550^***^	−0.0197	−0.0057	−0.0128
	(−2.7043)	(−0.3840)	(−3.1322)	(−0.8781)	(−0.1768)	(−0.4120)
Smoking	−0.1984^***^	−0.2262^***^	0.0756	−0.3369^***^	−0.3630^***^	−0.0019
	(−3.6375)	(−4.0555)	(0.3318)	(−4.0755)	(−4.3155)	(−0.0053)
Drinking	0.0923^**^	0.0955^**^	0.1896	0.0118	0.0304	0.3250
	(2.1749)	(2.1302)	(1.4309)	(0.1564)	(0.3816)	(1.3551)
Family size	−0.0275^***^	−0.0291^**^	−0.0255^*^	−0.0514^***^	−0.0652^***^	−0.0328
	(−2.8854)	(−2.3397)	(−1.7295)	(−3.3932)	(−3.0940)	(−1.5036)
Household income	0.0449^**^	0.0885^***^	0.0072	0.1393^***^	0.2468^***^	0.0316
	(2.4249)	(2.6941)	(0.3389)	(4.5897)	(5.1583)	(0.8072)
Engel coefficient	0.0232	0.1331	−0.0671	−0.0624	−0.3054	0.2454
	(0.2732)	(1.0814)	(−0.5726)	(−0.3536)	(−1.2244)	(0.9919)
Number of children	−0.0606^*^	−0.0586	−0.0675	−0.0384	−0.0254	−0.0506
	(−1.7992)	(−1.2848)	(−1.3829)	(−0.9331)	(−0.4550)	(−0.8383)
Year fixed effect	YES	YES	YES	YES	YES	YES
Province fixed effect	YES	YES	YES	YES	YES	YES
Age-group fixed effect	YES	YES	YES	YES	YES	YES
*N*	27,298	13,292	14,006	13,265	6,552	6,713
*R* ^2^	0.0733	0.0742	0.0871	0.0721	0.0647	0.0823

Coefficients are reported with Z-statistics in parentheses. ^*^p < 0.10, ^**^p < 0.05, ^***^p < 0.01.

**Alternative model specification**. To rule out potential confounding from time-varying provincial-level unobservable factors, such as regional economic fluctuations, local policy changes, and public health events—we further adopted a province–year fixed effects model as a robustness check, in place of the separate province and year fixed effects used in the main specification.

Table 5 reports the estimation results, and the core findings remain robust. For the full sample, Column 1 shows that the linear term of marriage duration is positive and statistically significant (β = −0.0403, *p* < 0.01), while the quadratic term is negative and statistically significant (β = −0.0009, *p* < 0.01), indicating that the inverted *U*-shaped relationship between MD and BMI still holds. A gender comparison reveals that the absolute magnitude of both the linear and quadratic coefficients is larger for men than for women, consistent with the pattern observed in the main analysis. Overall, our core conclusions remain robust after more stringently controlling for province–year specific time-varying confounding factors.

**Table 5:** Robustness test: alternative model specification.

	BMI		
Variables	All	Men	Women
	(1)	(2)	(3)
Marriage duration (MD)	0.0403^***^	0.0496^***^	0.0467^***^
	(5.0501)	(4.3874)	(4.1616)
MD^2^	−0.0009^***^	−0.0012^***^	−0.0010^***^
	(−4.4441)	(−3.8734)	(−3.7926)
Gender	0.8524^***^		
	(12.5271)		
Age	0.0172^***^	0.0021	0.0306^***^
	(2.6115)	(0.2339)	(3.2435)
Education	−0.0055	0.0549^***^	−0.0455^***^
	(−0.7242)	(4.9596)	(−4.3152)
Health	−0.0395^***^	−0.0088	−0.0623^***^
	(−3.0338)	(−0.4667)	(−3.4799)
Smoking	−0.1624^***^	−0.1830^***^	0.0573
	(−2.9585)	(−3.2743)	(0.2366)
Drinking	0.0714^*^	0.0689	0.1570
	(1.7051)	(1.5190)	(1.4083)
Family size	−0.0280^***^	−0.0330^***^	−0.0251^*^
	(−2.9432)	(−2.6896)	(−1.6809)
Household income	0.0589^***^	0.1168^***^	0.0081
	(3.6688)	(4.7595)	(0.3750)
Engel coefficient	−0.0143	0.0927	−0.0990
	(−0.1678)	(0.7654)	(−0.8331)
Number of children	−0.0721^**^	−0.0560	−0.0936^*^
	(−2.1380)	(−1.2182)	(−1.9043)
Province–Year fixed effect	YES	YES	YES
Age-group fixed effect	YES	YES	YES
*N*	29,734	14,497	15,237
*R* ^2^	0.0739	0.0738	0.0863

Coefficients are reported with Z-statistics in parentheses. ^*^p < 0.10, ^**^p < 0.05, ^***^p < 0.01.

**Instrumental variables approach**. To address endogeneity concerns regarding marriage duration, we used the relative age at first marriage as an instrumental variable, defined as the difference between an individual's actual age at first marriage and the provincial average in 2010. Data on the provincial average age at first marriage by sex in 2010 were obtained from the Major Figures on 2010 Population Census of China. The OLS estimation of marriage duration may suffer from two sources of endogeneity. First, omitted variable bias may arise because individual personality traits, such as conscientiousness and impulsivity, as well as early-life family background, could simultaneously affect both the age at first marriage and adult BMI, making it difficult for OLS to isolate the net effect of marriage. Second, reverse causality may exist, as an individual's current weight status could influence marital satisfaction or partner relationship quality, which in turn may affect the duration of marriage.

In terms of relevance, for married individuals observed at a given survey wave, a larger relative age at first marriage (i.e., marrying later) mechanically implies a shorter marriage duration at the time of the survey. Moreover, a larger relative age at first marriage indicates a greater deviation from the socially normative age at marriage, often referred to as the “social clock,” which is associated with weaker marital stability (65, 66), further reducing the observed marriage duration. These two mechanisms reinforce the negative IV-endogenous relationship.

Regarding the exclusion restriction, the provincial average age at first marriage in 2010 predates the individual survey waves from 2014 onward, which mitigates concerns about reverse causality to some extent. It is worth noting, however, that we do not claim that an individual's age at first marriage is entirely unrelated to education, socioeconomic status, birth cohort, or personality traits. Nevertheless, by subtracting the provincial mean and incorporating a rich set of covariates at both the individual and household levels, including education, health behaviors, household economic conditions, and number of children, among others, and crucially, by introducing age-group fixed effects, the identifying variation in our strategy is largely confined to the “deviation” of an individual's age at first marriage relative to peers. This comparison is made within the same survey year, province, age group, and socioeconomic background. Under this specification, the residual variation in this “deviation” primarily reflects the influence of social normative pressures on individual marital trajectories. More specifically, deviation from the social clock may indirectly affect BMI by reducing marital stability and marital quality, which in turn alter couples' shared lifestyle patterns (23, 67). We acknowledge that unobserved factors, including personality traits, may still pose potential threats to the IV estimates. Therefore, we treat the IV results as supplementary evidence for robustness checks rather than as definitive causal identification.

To address endogeneity of the quadratic marriage duration term, we follow Wooldridge (68) to construct non-linear instruments. We first regress marriage duration on the relative age at first marriage to obtain fitted values, then use their squared term as the instrument for marriage duration squared.

Table 6 presents the instrumental variable estimates. The Kleibergen-Paap rk LM (4,497.432, *p* < 0.01) statistic rejects the null of underidentification. The Kleibergen-Paap rk Wald F statistic is well above the Stock-Yogo 10% critical value of 16.38, ruling out concerns over weak instruments. Collectively, these diagnostics support the validity of the chosen instruments. The first-stage coefficient on the IV (relative age at first marriage) is negative and significant (β = −0.8612, *p* < 0.01). Furthermore, the IV estimates are qualitatively consistent with the baseline results, reinforcing the robustness of our core findings.

**Table 6:** Robustness test: instrumental variables approach.

Variables	BMI
	IV
$\tilde{\text{MD}}$	0.1074^***^
	(4.7875)
${\tilde{\text{MD}}}^{2}$	−0.0014^***^
	(−3.0935)
Gender	0.9991^***^
	(19.4077)
Age	−0.0200^**^
	(−2.2637)
Education	−0.0047
	(−0.8173)
Health	−0.0732^***^
	(−4.1258)
Smoking	−0.3059^***^
	(−5.5598)
Drinking	−0.0009
	(−0.0153)
Family size	−0.0357^***^
	(−3.0544)
Household income	0.1523^***^
	(6.3195)
Engel coefficient	0.1888
	(1.3852)
Number of children	−0.0677^**^
	(−2.3499)
Year fixed effect	Yes
Province fixed effect	Yes
Age-group fixed effect	Yes
Kleibergen-Paap rk LM statistic	4,497.432
Kleibergen-Paap rk Wald F statistic	4,738.915
Stock-Yogo 10% maximal IV size	7.03
*N*	29,734
*R* ^2^	0.0736

Coefficients are reported with Z-statistics in parentheses. ^*^p < 0.10, ^**^p < 0.05, ^***^p < 0.01.

### 4.3 Heterogeneity analysis

#### 4.3.1 Non-parents vs. parents

As discussed above, women's post–marital weight changes are shaped by two countervailing forces—domestic role expectations and aesthetic pressures—resulting in a net effect that is relatively weak and unstable. This suggests that weight changes among married women may not be directly driven by marital status *per se*, but rather by the accompanying transformation in family roles, particularly the assumption of the “mother” role. We therefore proceed to conduct a heterogeneity analysis by parenthood status.

Table 7 reports the heterogeneity analysis results by parenthood status. Among parents, Columns 4–6 show that the linear term of marriage duration is positive and significant, while the quadratic term is negative and significant, confirming an inverted U-shaped relationship for the full sample. In contrast, among non-parents, the inverted *U*-shaped relationship remains significant only for men (Column 2), while no clear inverted *U*-shaped pattern emerges for the full sample (Column 1) or for women (Column 3).

**Table 7:** Heterogeneity analysis: non-parents vs. parents.

	Non-parents			Parents		
Variables	All	Men	Women	All	Men	Women
	(1)	(2)	(3)	(4)	(5)	(6)
Marriage duration (MD)	0.1188	0.1915^**^	0.0701	0.0715^***^	0.0758^***^	0.0723^***^
	(1.3539)	(2.0884)	(0.5106)	(7.9950)	(6.8518)	(6.2001)
MD^2^	−0.0025	−0.0068^***^	0.0007	−0.0014^***^	−0.0015^***^	−0.0014^***^
	(−0.9530)	(−3.0005)	(0.1881)	(−6.7970)	(−5.9021)	(−5.5511)
Gender	1.4762^***^			0.8785^***^		
	(2.8614)			(13.0748)		
Age	−0.0545	−0.0289	−0.1447	0.0044	−0.0118	0.0166
	(−0.7683)	(−0.3173)	(−1.1600)	(0.5816)	(−1.2151)	(1.5605)
Education	−0.0867	0.0331	−0.1477^*^	−0.0036	0.0542^***^	−0.0390^***^
	(−1.5399)	(0.4522)	(−1.6829)	(−0.4712)	(4.9494)	(−3.9128)
Health	−0.2013	0.1659	−0.4046^**^	−0.0327^***^	−0.0114	−0.0479^***^
	(−1.4945)	(0.7228)	(−2.2612)	(−2.6522)	(−0.6319)	(−3.0948)
Smoking	−0.4609	0.1564	−2.0293^*^	−0.1766^***^	−0.2024^***^	0.1191
	(−0.9317)	(0.3106)	(−1.6651)	(−3.3600)	(−3.7749)	(0.6620)
Drinking	0.3661	0.3038	1.6669	0.0832^**^	0.0959^**^	0.1206
	(0.7360)	(0.5897)	(1.2707)	(2.0870)	(2.2474)	(1.1253)
Family size	−0.1524	−0.0541	−0.1901	−0.0236^***^	−0.0314^***^	−0.0145
	(−1.2334)	(−0.3105)	(−1.0670)	(−2.5948)	(−2.6392)	(−1.0785)
Household income	0.1023	0.1619	0.0385	0.0385^**^	0.0806^**^	0.0019
	(1.2921)	(1.2660)	(0.3343)	(2.1388)	(2.5471)	(0.0965)
Engel coefficient	−1.4941^*^	−0.8437	−1.8023^*^	0.0266	0.1474	−0.0751
	(−1.8605)	(−0.7503)	(−1.6679)	(0.3322)	(1.2773)	(−0.6861)
Number of children				−0.0685^**^	−0.0701	−0.0730^*^
				(−2.0783)	(−1.5326)	(−1.6976)
Year fixed effect	YES	YES	YES	YES	YES	YES
Province fixed effect	YES	YES	YES	YES	YES	YES
Age-group fixed effect	YES	YES	YES	YES	YES	YES
*N*	498	247	251	29,236	14,250	14,986
*R* ^2^	0.1741	0.1675	0.3179	0.0739	0.0717	0.0888

Coefficients are reported with Z-statistics in parentheses. ^*^p < 0.10, ^**^p < 0.05, ^***^p < 0.01.

These findings suggest that the presence of children is closely associated with the strength of the inverted *U*-shaped relationship between marriage and BMI. Among fathers, the marriage duration–BMI association exhibits a relatively moderate inverted *U*-shaped trajectory. By contrast, childless men display a more substantial BMI increase in the initial phase of marriage, a pattern consistent with the honeymoon effect. For childless women, the cumulative effect of marriage on weight is the least pronounced among all groups. One possible interpretation is that childless women are less subject to parenting-related time constraints and role burdens. It should be noted, however, that the non-parent subsample consists of only 498 observations (247 men and 251 women), considerably smaller than the parent subsample of 29,236. This imbalance primarily reflects the demographic reality that married individuals without children are a minority in the Chinese context. Consequently, the statistical power for the non-parent group is relatively limited, and these findings should be interpreted as suggestive evidence requiring further validation in future research.

#### 4.3.2 Rural vs. urban

Significant differences exist between urban and rural residents in China in terms of physical activity patterns, income levels, and food consumption structures. These factors may generate differential effects of marriage on body weight across urban and rural contexts (69). For example, regarding dietary patterns, rural residents generally have lower dietary diversity and a more homogeneous dietary structure compared to urban residents. According to statistics from the National Bureau of Statistics of China, the per capita consumption of meat, aquatic products, eggs and milk in urban areas generally exceeds that of rural residents, implying a narrower dietary base in rural settings and thus greater potential for post-marital dietary shifts. Thus, we further explored the effects of urban and rural marriage on body weight. We divided them into six groups according to urban/rural residence and gender: rural (aggregate), rural males, rural females, urban (aggregate), urban males, and urban females.

Table 8 presents the regression results by urban and rural residence. First, the inverted *U*-shaped relationship is observed in both the rural (Column 1) and urban (Column 4) samples, with the coefficient for the rural sample being 56.60% larger than that for the urban sample. With respect to gender differences, the positive association between MD and BMI is stronger for women than for men in rural areas, whereas in urban areas, it is stronger for men than for women. Same-gender comparisons show that the association is stronger for rural individuals than for urban individuals in both men and women. The stronger association observed in rural areas may partly reflect differential dietary transitions after marriage. Chinese evidence indicates that living with a spouse is positively associated with dietary diversity, with a particularly pronounced effect in rural areas (70). This marital dietary effect may translate into more pronounced weight changes, especially given the ongoing nutrition transition in rural China. Taken together, one plausible interpretation is that marriage-related improvements in dietary habits, if present, might yield greater marginal health returns in rural areas, where baseline nutritional conditions are relatively poor. This could help explain the more pronounced marriage–BMI association observed among rural respondents.

**Table 8:** Heterogeneity analysis: rural vs. urban.

	Rural			Urban		
Variables	All	Men	Women	All	Men	Women
	(1)	(2)	(3)	(4)	(5)	(6)
Marriage duration (MD)	0.0830^***^	0.0761^***^	0.0991^***^	0.0530^***^	0.0728^***^	0.0434^**^
	(6.7695)	(4.5819)	(4.5819)	(4.3967)	(4.4092)	(2.4789)
MD^2^	−0.0016^***^	−0.0015^***^	−0.0019^***^	−0.0011^***^	−0.0013^***^	−0.0010^***^
	(−5.2130)	(−3.8190)	(−3.8190)	(−3.7803)	(−3.1787)	(−2.9268)
Gender	0.7076^***^			1.1682^***^		
	(7.6799)			(13.0978)		
Age	−0.0101	−0.0235^*^	0.0011	0.0196^**^	−0.0105	0.0477^***^
	(−0.9652)	(−1.7539)	(0.0702)	(2.0057)	(−0.8172)	(3.2824)
Education	0.0046	0.0571^***^	−0.0328^**^	−0.0242^**^	0.0341^**^	−0.0607^***^
	(0.4490)	(3.9678)	(−2.2446)	(−2.2574)	(2.1495)	(−4.1742)
Health	−0.0187	0.0234	−0.0491^**^	−0.0536^***^	−0.0574^**^	−0.0477^**^
	(−1.0574)	(0.9249)	(−1.9943)	(−3.0225)	(−2.1926)	(−1.9949)
Smoking	−0.2665^***^	−0.2777^***^	−0.1893	−0.1002	−0.1381^*^	0.4216
	(−3.6380)	(−3.6482)	(−0.6906)	(−1.3565)	(−1.8436)	(1.1995)
Drinking	0.0723	0.0987	−0.0064	0.0892	0.0809	0.2895^**^
	(1.2073)	(1.5755)	(−0.0322)	(1.5574)	(1.2920)	(2.0459)
Family size	−0.0316^**^	−0.0200	−0.0407^**^	−0.0099	−0.0416^**^	0.0210
	(−2.5051)	(−1.2148)	(−2.0955)	(−0.6829)	(−2.0635)	(1.0326)
Household income	0.0590^***^	0.1261^***^	−0.0050	0.0110	0.0209	0.0022
	(2.6614)	(3.7737)	(−0.1665)	(0.3611)	(0.3493)	(0.0759)
Engel coefficient	−0.2014^*^	−0.0132	−0.3648^**^	0.1782	0.1474	0.2311
	(−1.6515)	(−0.0762)	(−2.1363)	(1.6019)	(0.8959)	(1.5335)
Number of children	−0.0885^**^	−0.0568	−0.1308^**^	−0.0065	−0.0311	0.0149
	(−2.0222)	(−0.9697)	(−2.0355)	(−0.1321)	(−0.4669)	(0.2098)
Year fixed effect	YES	YES	YES	YES	YES	YES
Province fixed effect	YES	YES	YES	YES	YES	YES
Age-group fixed effect	YES	YES	YES	YES	YES	YES
*N*	14,875	7,344	7,531	14,859	7,153	7,706
*R* ^2^	0.0769	0.0773	0.1006	0.0903	0.0643	0.1055

Coefficients are reported with Z-statistics in parentheses. ^*^p < 0.10, ^**^p < 0.05, ^***^p < 0.01.

#### 4.3.3 North vs. south

The structure of agricultural production in Southern and Northern China creates differences in dietary preferences between the North and the South. The northern regions of China follow a wheat-based dietary pattern. The northern wheat-based dietary pattern (NWB) group had a higher carbohydrate intake, a lower vegetable intake, and the lowest protein intake from animal sources (only 20.3%). Conversely, the southern regions of China follow a rice-based dietary pattern. The southern rice-based dietary pattern (SRB) group had the highest intake of vegetables (particularly dark green vegetables and starchy vegetables), red meat, fish, and poultry. Compared to SRB, NWB showed a stronger positive association with overweight/obesity (71). Wang et al. (72) utilized data from the China Chronic Disease and Risk Factor Surveillance (CCDRFS) program to demonstrate that the average BMI of individuals in Northern China is 2 kg/m^2^ higher than that of individuals in Southern China, and the prevalence of obesity in Northern China is more than three times greater than in Southern China. Considering the importance of dietary differences in northern and southern China on body weight, we further explored the difference in the influence of marriage on body weight between northern and southern residents. We divided them into six groups according to northern/southern residence and gender: Southern (aggregate), Southern males, Southern females, Northern (aggregate), Northern males, and Northern females.

Table 9 presents the estimation results by region. Columns 1 and 4 indicate that the inverted *U*-shaped relationship between marriage duration and BMI is statistically significant in both the southern and northern subsamples, with the linear coefficient for the northern sample being 29.20% larger than that for the southern sample. With respect to gender differences, the positive marriage-BMI association is slightly weaker among women than among men across both regions. Same-gender comparisons further demonstrate that the linear coefficient for northern men (β = 0.0829) is markedly higher than that for southern men (β = 0.0645), and the coefficient for northern women (β = 0.0806) exceeds that for southern women (β = 0.0640). These regional and gender disparities in the marriage–BMI association are likely rooted in fundamental differences in dietary patterns and nutritional intake between Northern and Southern China. In Northern China, where the baseline diet already skews toward high-carbohydrate and low-animal-protein composition, marriage-induced increases in caloric consumption and dietary diversity may exacerbate pre-existing nutritional imbalances, yielding larger marginal effects on energy surplus and BMI. In contrast, the Southern diet's greater baseline diversity and higher vegetable/protein intake may buffer against the weight-gain effects of post-marital dietary changes.

**Table 9:** Heterogeneity analysis: south vs. north.

	South			North		
Variables	All	Men	Women	All	Men	Women
	(1)	(2)	(3)	(4)	(5)	(6)
Marriage duration (MD)	0.0572^***^	0.0645^***^	0.0640^***^	0.0739^***^	0.0829^***^	0.0806^***^
	(4.3260)	(3.6985)	(3.2349)	(6.3514)	(5.1411)	(4.7736)
MD^2^	−0.0013^***^	−0.0015^***^	−0.0014^***^	−0.0014^***^	−0.0016^***^	−0.0015^***^
	(−4.1321)	(−3.8333)	(−2.9414)	(−5.2709)	(−4.3594)	(−4.1644)
Gender	0.9469^***^			0.8252^***^		
	(9.0290)			(9.6018)		
Age	0.0114	−0.0028	0.0257	0.0035	−0.0143	0.0185
	(1.0459)	(−0.1918)	(1.6093)	(0.3614)	(−1.1483)	(1.2232)
Education	0.0041	0.0622^***^	−0.0406^**^	−0.0089	0.0484^***^	−0.0402^***^
	(0.3531)	(3.8343)	(−2.4963)	(−0.9072)	(3.3396)	(−2.9839)
Health	0.0034	0.0280	−0.0166	−0.0569^***^	−0.0339	−0.0727^***^
	(0.1733)	(1.0199)	(−0.6026)	(−3.6018)	(−1.4433)	(−3.4109)
Smoking	−0.0759	−0.0559	0.0010	−0.2386^***^	−0.2847^***^	0.0560
	(−0.8909)	(−0.6311)	(0.0028)	(−3.5826)	(−4.2126)	(0.2024)
Drinking	−0.0119	−0.0096	0.0670	0.1700^***^	0.1684^***^	0.3322^**^
	(−0.1925)	(−0.1442)	(0.4039)	(3.1542)	(2.9418)	(2.0644)
Family size	−0.0039	−0.0133	0.0081	−0.0384^***^	−0.0422^**^	−0.0344^*^
	(−0.2921)	(−0.7409)	(0.4098)	(−3.0083)	(−2.5691)	(−1.7362)
Household income	0.0269	0.0318	0.0212	0.0556^***^	0.1292^***^	−0.0076
	(0.8874)	(0.5447)	(0.7279)	(2.5815)	(3.7957)	(−0.2691)
Engel coefficient	0.1604	0.3288^*^	0.0037	−0.0919	−0.0198	−0.1428
	(1.3379)	(1.9429)	(0.0215)	(−0.8538)	(−0.1256)	(−0.9759)
Number of children	−0.1035^**^	−0.1263^*^	−0.0860	−0.0428	−0.0171	−0.0750
	(−2.1218)	(−1.9325)	(−1.1952)	(−0.9754)	(−0.2851)	(−1.1863)
Year fixed effect	YES	YES	YES	YES	YES	YES
Province fixed effect	YES	YES	YES	YES	YES	YES
Age-group fixed effect	YES	YES	YES	YES	YES	YES
*N*	11,521	5,746	5,775	1,8213	8,751	9,462
*R* ^2^	0.0480	0.0485	0.0430	0.0674	0.0682	0.0846

Coefficients are reported with Z-statistics in parentheses. ^*^p < 0.10, ^**^p < 0.05, ^***^p < 0.01.

## 5 The moderating role of marital satisfaction

Living in a loving family with high marital quality, people tend to have less psychological stress, which is simultaneously associated with lower health-risk behaviors and a healthy weight (73). Considering the importance of relationship quality in marriage, we used marital satisfaction as a moderating variable to examine its effect on the relationship between marriage and weight.

Table 10 reports the moderating effect of marital satisfaction. The results show that this moderating effect exhibits significant gender heterogeneity: it is significant for men but not for women. Specifically, for the male subsample (Column 2), the interaction term between marital satisfaction and the linear term of marriage duration is negative and significant (β = −0.0028, *p* < 0.05), indicating that higher marital satisfaction weakens the positive marriage duration–BMI association. In contrast, for the female subsample (Column 3), none of the interaction terms are statistically significant, suggesting that marital satisfaction is not a key moderating factor in the relationship between marriage duration and BMI among women.

**Table 10:** The power of love: the moderating role of marital satisfaction.

	BMI		
Variables	All	Men	Women
	(1)	(2)	(3)
Marriage duration (MD_center)	0.0109^*^	0.0117^**^	0.0110
	(1.8570)	(2.4567)	(1.1904)
MD_center^2^	−0.0017^***^	−0.0020^***^	−0.0015^***^
	(−6.6129)	(−5.4577)	(−5.0753)
Marriage satisfaction (MS_center)	0.0404^**^	0.0420^*^	0.0381
	(2.0430)	(1.7531)	(1.4732)
MS_center^*^MD_center	−0.0032^**^	−0.0028^**^	−0.0026
	(−2.4036)	(−2.1894)	(−1.5457)
MS_center^*^MD_center^2^	−0.0001	−0.0002	−0.0000
	(−0.9359)	(−1.2320)	(−0.1770)
Gender	0.8704^***^		
	(12.9615)		
Age	0.0061	−0.0123	0.0214^*^
	(0.8248)	(−1.2957)	(1.8899)
Education	−0.0041	0.0532^***^	−0.0406^***^
	(−0.5428)	(4.8772)	(−3.8847)
Health	−0.0370^***^	−0.0090	−0.0580^***^
	(−3.0085)	(−0.4996)	(−3.4468)
Smoking	−0.1803^***^	−0.1992^***^	0.0506
	(−3.4514)	(−3.7254)	(0.2309)
Drinking	0.0920^**^	0.0976^**^	0.1711
	(2.2806)	(2.2640)	(1.4829)
Family size	−0.0266^***^	−0.0315^***^	−0.0205
	(−2.9304)	(−2.6415)	(−1.4765)
Household income	0.0423^**^	0.0864^***^	0.0039
	(2.4120)	(2.8178)	(0.1904)
Engel coefficient	−0.0020	0.1097	−0.0958
	(−0.0243)	(0.9450)	(−0.8561)
Number of children	−0.0653^**^	−0.0599	−0.0771
	(−2.0062)	(−1.3584)	(−1.6302)
Year fixed effect	YES	YES	YES
Province fixed effect	YES	YES	YES
Age-group fixed effect	YES	YES	YES
*N*	29,734	14,497	15,237
*R* ^2^	0.0748	0.0716	0.0887

Coefficients are reported with Z-statistics in parentheses. ^*^p < 0.10, ^**^p < 0.05, ^***^p < 0.01.

To rule out potential reverse causality, we further regressed current marital satisfaction on lagged BMI among men. The coefficient was not statistically significant (β = −0.0508, *p* = 0.372), further supporting the robustness of the moderating effect of marital satisfaction in the male subsample.

## 6 Discussion

This study uses unbalanced panel data from 29,734 observations in the CFPS (2014, 2018, 2020, 2022) to examine the association between marriage duration and BMI. The findings demonstrate a positive association between marriage and BMI. This result echoes the viewpoints of many scholars (13, 14). Distinct from prior research, this study reveals an inverted *U*-shaped relationship between MD and BMI. By integrating the health capital model and the Double ABC-X model family stress adaptation framework, this study systematically reveals the non-linear dynamic mechanism between marriage duration and weight change. It provides a plausible theoretical explanation for the contradictory conclusions found in existing studies, such as “weight gain in marriage,” “weight loss in marriage” and “marriage has nothing to do with weight” (7, 14, 74, 75).

The findings also suggest that the association between marriage duration and BMI differs significantly by gender. In particular, the positive association is stronger for men than for women, which aligns with findings from some earlier studies (14, 52). This further supports the gender role theory that men are more dependent on their spouses as “gatekeepers” of their health (40). Regarding parenthood differences, the inverted *U*-shaped relationship between marriage duration and BMI remains robust among parents, whereas among non-parents, a significant non-linear association is observed only for men. This pattern suggests that parental roles may influence the strength of the marriage-weight association through changes in time allocation and family responsibilities. In terms of regional differences, the positive marriage-BMI association is stronger in rural and northern areas. These regional disparities may reflect differences in dietary diversity and nutritional baselines across regions. In rural areas, premarital dietary patterns tend to be more homogeneous, which may create greater potential for post-marital dietary improvements and thus larger marginal effects on BMI. In northern China, the wheat-based dietary pattern is characterized by higher carbohydrate intake and lower animal protein consumption, which has been linked to a higher obesity risk (71). Future research could further explore the interplay between regional food environments and marital health effects.

In addition, the study found some interesting results. Marital satisfaction significantly moderates the inverted *U*-shaped relationship between marriage duration and BMI among men. Specifically, in the male subsample, the interaction term between marital satisfaction and the linear term of marriage duration is negative and significant. Higher marital satisfaction buffers the positive association between marriage duration and BMI among men. A possible explanation is that, couples with higher marital satisfaction may shift their lifestyle synchrony from shared eating behaviors to joint health-promoting behaviors, such as exercising together and monitoring each other's diets, which in turn facilitates weight loss (11, 12). Notably, this moderating effect is pronounced only among men, further highlighting gender differences in the health correlates of marriage.

This study expands the research on marriage and health by analyzing the relationship between marriage duration, marital quality and BMI, but the study still has some limitations. Firstly, this study is based on BMI as the sole indicator of adiposity. Although BMI is widely used for its simplicity and cross-population comparability, it does not capture fat distribution—particularly visceral or abdominal adiposity, which is more strongly linked to metabolic risk. Among middle-aged and older adults, age-related declines in lean muscle mass often coincide with increases in central fat accumulation, leading to a phenomenon known as “normal-weight obesity” or metabolically obese normal weight. Consequently, using BMI alone may attenuate the observed association between marital factors and underlying metabolic health risks. In addition, given that CFPS relies on self-reported anthropometric measures, the resulting measurement error and reporting bias in BMI warrant caution when interpreting the effect sizes. Secondly, this study fitted an inverted *U*-shaped relationship between marriage duration and BMI based on the quadratic term, ignoring the repeated fluctuations in weight status (among normal, overweight, and obese). Future studies may draw on group-based trajectory models (GBTM) or Markov state transition models to more finely delineate the dynamic patterns of body weight within marriage. Thirdly, marital quality is captured only by subjective satisfaction, omitting other key dimensions such as conflict, support, and dyadic coping. The cross-sectional nature of satisfaction and BMI measurements precludes causal ordering. While lagged BMI analysis found no evidence of longitudinal reverse causality, contemporaneous bidirectionality cannot be excluded. Future multi-wave panel studies are needed to clarify the causal dynamics between marital satisfaction and weight trajectories. Fourthly, the Double ABC-X constructs (stressors, resources, and cognitive appraisals) were not directly measured in our data. Future studies could directly include such measures to clarify the underlying psychological mechanisms. Fifth, the requirement of at least two interviews introduces panel attrition, which may bias our estimates. Given our limited observational window, future studies with extended longitudinal designs and more frequent follow-ups are needed to better assess and correct for this selection bias.

## 7 Conclusion

Obesity is a rising epidemic in China and has become a major issue for public health and socio-economic development. Most existing studies have approached the issue from physiological and medical perspectives. However, marital relationships, as a central institutional environment in individuals' lives, exert a profound influence on daily dietary patterns, physical activity routines, and health behavior norms. These factors are closely tied to individual obesity risk. From a dynamic perspective centered on marriage duration, this study reinterprets the association between marriage and body weight as a rational adaptive process. This process involves the accumulation of family pressure, resource mobilization, and cognitive adjustment over time. Integrating the health capital model with the Double ABC-X family stress adaptation framework, we propose that the association between marriage and body weight is not a linear accumulation, but rather a dynamic process that evolves non-linearly over the course of the relationship.

Empirical findings indicate an inverted *U*-shaped association between marriage duration and BMI. Notably, this association is significantly stronger in men than in women. The inverted *U*-shaped relationship between marriage duration and BMI becomes more robust after the transition to parenthood. In addition, significant regional differences are observed: marriage is associated with higher BMI in rural and northern areas. Furthermore, the moderating effect of marital satisfaction buffers the positive association between marriage duration and BMI among men. The study provides a novel paradigm for understanding how marital relationships shape healthy behaviors. It also lays an empirical foundation for promoting obesity prevention and control strategies that take the family as a unit and take into account gender and regional sensitivity.

## Data Availability

Publicly available datasets were analyzed in this study. The data from the China Family Panel Studies (CFPS) is open to the public and can be obtained free of charge through this website (https://cfpsdata.pku.edu.cn/#/home).

## References

[1] Risk Factor Collaboration NCD-RisC NCD-RisC Worldwide trends in underweight and obesity from 1990 to 2022: a pooled analysis of 3663 population-representative studies with 222 million children adolescents and adults. Lancet. (2024) 403:1027–50. doi: 10.1016/S0140-6736(23)02750-2 38432237 PMC7615769

[2] World Health Organization. Obesity and Overweight: Key Facts (2024). Available online at: https://www.who.int/news-room/fact-sheets/detail/obesity-and-overweight (Accessed January 9, 2026).

[3] World Obesity Federation. World Obesity Atlas 2023. London: World Obesity Federation (2023). p. 72.

[4] Bowen M. Family Therapy in Clinical Practice. New York, NY: Jason Aronson (1978).

[5] Sobal J. Marriage, obesity and dieting. Marriage Fam Rev. (1984) 7:115–39. doi: 10.1300/J002v07n01_10

[6] Anson O. Marital status and women's health revisited: the importance of a proximate adult. J Marriage Fam. (1989) 51:185. doi: 10.2307/352379

[7] Averett SL Sikora A Argys LM. For better or worse: relationship status and body mass index. Econ Hum Biol. (2008) 6:330–49. doi: 10.1016/j.ehb.2008.07.003 18753018

[8] Falba TA Sindelar JL. Spousal concordance in health behavior change. Health Serv Res. (2008) 43:96–116. doi: 10.1111/j.1475-6773.2007.00754.x 18211520 PMC2323137

[9] Lim S Raymo JM. Marriage and women's health in Japan. J Marriage Fam. (2016) 78:780–96. doi: 10.1111/jomf.12298 30774150 PMC6377079

[10] Spoor STP Bekker MHJ Van Strien T van Heck GL. Relations between negative affect, coping, and emotional eating. Appetite. (2007) 48:368–76. doi: 10.1016/j.appet.2006.10.005 17145096

[11] Markey CN Markey PM Gray HF. Romantic relationships and health: an examination of individuals' perceptions of their romantic partners' influences on their health. Sex Roles. (2007) 57:435–45. doi: 10.1007/s11199-007-9266-5

[12] Theiss JA Carpenter AM Leustek J. Partner facilitation and partner interference in individuals' weight loss goals. Qual Health Res. (2016) 26:1318–30. doi: 10.1177/1049732315583980 25904678

[13] Teachman J. Body weight, marital status, and changes in marital status. J Fam Issues. (2016) 37:74–96. doi: 10.1177/0192513X13508404 26778872 PMC4714799

[14] Liu J Garstka MA Chai Z Chen Y Lipkova V Cooper ME . Marriage contributes to higher obesity risk in China: findings from the China health and nutrition survey. Ann Transl Med. (2021) 9:564. doi: 10.21037/atm-20-4550 33987262 PMC8105842

[15] Novak JR Pratt K Hernandez DC Berge JM. Family systems and obesity: a review of key concepts and influences within and between family subsystems and a call for family-informed interventions. J Fam Theory Rev. (2023) 15:38–56. doi: 10.1111/jftr.12474

[16] Quan S Zhang H. The relationship between marriage and body mass index in China: evidence from the China health and nutrition survey. Econ Hum Biol. (2024) 53:101368. doi: 10.1016/j.ehb.2024.101368 38368723

[17] Grossman M. On the concept of health capital and the demand for health. J Polit Econ. (1972) 80:223–55. doi: 10.1086/259880

[18] McCubbin HI Patterson JM. The family stress process: the double ABCX model of adjustment and adaptation. Marriage Fam Rev. (1983) 6:7–37.

[19] Umberson D Karas Montez J. Social relationships and health: a flashpoint for health policy. J Health Soc Behav. (2010) 51:S54–66. doi: 10.1177/0022146510383501 20943583 PMC3150158

[20] Jeffery RW Rick AM. Cross-sectional and longitudinal associations between body mass index and marriage-related factors. Obes Res. (2002) 10:809–15. doi: 10.1038/oby.2002.109 12181390

[21] Civi RM Mehranfar S Plunkett R Veenstra G Conklin AI. A systematic review of social connections as determinants of obesity: longitudinal evidence limited to marital transitions. Obes Rev. (2024) 25:e13819. doi: 10.1111/obr.13819 39159971

[22] Nomaguchi KM Bianchi SM. Exercise time: gender differences in the effects of marriage, parenthood, and employment. J Marriage Fam. (2004) 66:413–30. doi: 10.1111/j.1741-3737.2004.00029.x

[23] Meltzer AL McNulty JK Karney BR. Social support and weight maintenance in marriage: The interactive effects of support seeking, support provision, and gender. J Fam Psychol. (2012) 26:678–87. doi: 10.1037/a0029364 22866928 PMC4112742

[24] Irani L Vemireddy V. Getting the measurement right! Quantifying time poverty and multitasking from childcare among mothers with children across different age groups in rural north India. Asian Popul Stud. (2020) 17:1–23. doi: 10.1080/17441730.2020.1778854

[25] Umberson D Liu H Mirowsky J Reczek C. Parenthood and trajectories of change in body weight over the life course. Soc Sci Med. (2011) 73:1323–31. doi: 10.1016/j.socscimed.2011.08.014 21925781 PMC3391503

[26] Meltzer AL Novak SA McNulty JK Butler EA Karney BR. Marital satisfaction predicts weight gain in early marriage. Health Psychol. (2013) 32:824–7. doi: 10.1037/a0031593 23477578

[27] Tang F Pan YH Deng HL. Effect of marriage on overweight and obesity: evidence from China. BMC Public Health. (2024) 24:3591. doi: 10.1186/s12889-024-21184-3 39725877 PMC11674091

[28] Kent S Fusco F Gray A Jebb SA Cairns BJ Mihaylova B . Body mass index and healthcare costs: a systematic literature review of individual participant data studies. Obes Rev. (2017) 18:869–79. doi: 10.1111/obr.12560 28544197

[29] Wang YP Liu M Liu J. Association of body mass index with risk of household catastrophic health expenditure in China: a population-based cohort study. Nutrients. (2022) 14:4014. doi: 10.3390/nu14194014 36235667 PMC9571178

[30] Li C Li X Li YM Niu XR. The nonlinear relationship between body mass index (BMI) and perceived depression in the Chinese population. Psychol Res Behav Manag. (2023) 16:2103–24. doi: 10.2147/PRBM.S411112 37325255 PMC10263158

[31] Carels RA Miller JC Hlavka R Selensky J Shonrock AMT Ellis JM . Associations between husbands' weight bias and related concerns and husbands' and wives' psychological and relationship outcomes. Body Image. (2020) 35:11–21. doi: 10.1016/j.bodyim.2020.07.008 32798926

[32] Carels RA Miller JC Hlavka R Shonrock AMT. The interaction between BMI, weight criticism, weight bias, and psychological and relational outcomes within marriage. Eat Weight Disord. (2022) 27:1887–93. doi: 10.1007/s40519-021-01324-z 34704191

[33] Markey CN Gomel JN Markey PM. Romantic relationships and eating regulation: an investigation of partners' attempts to control each others' eating behaviors. J Health Psychol. (2008) 13:422–32. doi: 10.1177/1359105307088145 18420775

[34] Tang ST Yu SQ Wang CC Yang JG Gao LL Chen XM . Factors influence the acceptance of surgical treatment in Chinese bariatric surgery candidates. Obes Surg. (2018) 28:2767–73. doi: 10.1007/s11695-018-3237-5 29707748 PMC6132737

[35] Rong SC Yang NL Liang H Zang Y Hua HX. Mediating effect of perceived social support between adult attachment and self-management inpatients after bariatric and metabolic surgery. J Nurs. (2025) 32:51–5. doi: 10.16460/j.issn2097-6569.2025.13.051

[36] Vo TT Le QD. Application of the health belief model to study weight management behavioral intentions among adults in Ho Chi Minh City, Vietnam: a cross-sectional study. BMC Public Health. (2025) 25:2436. doi: 10.1186/s12889-025-23637-9 40646482 PMC12247397

[37] Corder K Winpenny EM Foubister C Guagliano JM Hartwig XM Love R . Becoming a parent: a systematic review and meta-analysis of changes in BMI, diet, and physical activity. Obes Rev. (2020) 21:e12959. doi: 10.1111/obr.12959 31955517 PMC7078970

[38] Cobb LK McAdams-DeMarco MA Gudzune KA Anderson CAM Demerath E Woodward M . Changes in Body mass index and obesity risk in married couples over 25 years: the ARIC cohort study. Am J Epidemiol. (2016) 183:435–43. doi: 10.1093/aje/kwv112 26405117 PMC4772434

[39] Bernard J. The Future of Marriage. New York, NY: World Publishing Times Mirror (1972).

[40] Umberson D. Gender, marital status and the social control of health behavior. Soc Sci Med. (1992) 34:907–17. doi: 10.1016/0277-9536(92)90259-S 1604380

[41] Waite LJ Lehrer EL. The benefits from marriage and religion in the United States: a comparative analysis. Popul Dev Rev. (2003) 29:255–75. doi: 10.1111/j.1728-4457.2003.00255.x 19129930 PMC2614329

[42] Cohen S. Social relationships and health. Am Psychol. (2004) 59:676–84. doi: 10.1037/0003-066X.59.8.676 15554821

[43] Hersch J Stratton LS. Housework, wages, and the division of housework time for employed spouses. Am Econ Rev. (1994) 84:120–5.

[44] Welch N McNaughton SA Hunter W Hume C Crawford D. Is the perception of time pressure a barrier to healthy eating and physical activity among women? Public Health Nutr. (2009) 12:888–95. doi: 10.1017/S1368980008003066 18647424

[45] Kalenkoski CM Hamrick KS. How does time poverty affect behavior? A look at eating and physical activity. Appl Econ Perspect Policy. (2013) 35:89–105. doi: 10.1093/aepp/pps034

[46] Kamp Dush CM Yavorsky JE Schoppe-Sullivan SJ. What are men doing while women perform extra unpaid labor? Leisure and specialization at the transition to parenthood. Sex Roles. (2018) 78:715–30. doi: 10.1007/s11199-017-0841-0 30013287 PMC6044284

[47] Sobal J. Social and cultural influences on obesity. In: Per Bjorntorp International textbook of obesity. Hoboken, NJ: Wiley (2001). pp 305–22.

[48] Bordo S. Unbearable Weight: Feminism, Western Culture, and the Body. Oakland, CA: University of California Press (2003).

[49] Roehling MV Roehling PV Pichler S. The relationship between body weight and perceived weight-related employment discrimination: the role of sex and race. J Vocat Behav. (2007) 71:300–18. doi: 10.1016/j.jvb.2007.04.008

[50] Lewis S Thomas SL Blood RW Castle DJ Hyde J Komesaroff PA . How do obese individuals perceive and respond to the different types of obesity stigma that they encounter in their daily lives? A qualitative study. Soc Sci Med. (2011) 73:1349–56. doi: 10.1016/j.socscimed.2011.08.021 21944718

[51] Jiang QC Zhang KZ. “Economics of Beauty” in China's labor market: does stature matter? China Econ Q. (2013) 12:983–1006. doi: 10.13821/j.cnki.ceq.2013.03.004

[52] Sobal J Rauschenbach BS Frongillo EA. Marital status, fatness and obesity. Soc Sci Med. (1992) 35:915–23. doi: 10.1016/0277-9536(92)90106-Z 1411692

[53] Bodenmann G. Dyadic coping: a systemic-transactional view of stress and coping among couples: theory and empirical findings. Eur Rev Appl Psychol (1997). 47:137–41.

[54] Revenson TA Kayser K Bodenmann G . Couples Coping with Stress: Emerging Perspectives on Dyadic Coping. Washington, DC: American Psychological Association (2005).

[55] Umberson D Williams K Powers DA Liu H Needham B. You make me sick: marital quality and health over the life course. J Health Soc Behav. (2006) 47:1–16. doi: 10.1177/002214650604700101 16583772 PMC3149975

[56] Kouvonen A Stafford M De Vogli R Shipley MJ Marmot MG Cox T . Negative aspects of close relationships as a predictor of increased body mass index and waist circumference: the whitehall II study. Am J Public Health. (2011) 101:1474–80. doi: 10.2105/AJPH.2010.300115 21680928 PMC3134502

[57] Kiecolt-Glaser JK Newton T Cacioppo JT MacCallum RC Glaser R Malarkey WB . Marital conflict and endocrine function: are men really more physiologically affected than women? J Consult Clin Psychol. (1996) 64:324–32. 8871417 10.1037//0022-006x.64.2.324

[58] Saxbe D Repetti RL. For better or worse? Coregulation of couples' cortisol levels and mood states. J Pers Soc Psychol. (2010) 98:92–103. doi: 10.1037/a0016959 20053034

[59] Robles TF Slatcher RB Trombello JM McGinn MM. Marital quality and health: a meta-analytic review. Psychol Bull. (2014) 140:140–87. doi: 10.1037/a0031859 23527470 PMC3872512

[60] Whisman MA Baucom DH. Intimate relationships and psychopathology. Clin Child Fam Psychol Rev. (2012) 15:4–13. doi: 10.1007/s10567-011-0107-2 22124792

[61] El-Sheikh M Kelly R Rauer A. Quick to berate, slow to sleep: interpartner psychological conflict, mental health, and sleep. Health Psychol. (2013) 32:1057–66. doi: 10.1037/a0031786 23544995 PMC4086421

[62] Pontzer H Yamada Y Sagayama H . Daily energy expenditure through the human life course. Science. (2021) 373:808–12. doi: 10.1530/ey.19.14.1 34385400 PMC8370708

[63] Aiken LS West SG. Multiple Regression: Testing and Interpreting Interactions. Newbury Park, CA: Sage Publications (1991).

[64] Lind JT Mehlum H. With or without U? The appropriate test for a U-shaped relationship. Oxf Bull Econ Stat. (2010) 72:109–18. doi: 10.1111/j.1468-0084.2009.00569.x

[65] Wolfinger NH. Want to Avoid Divorce? Wait to Get Married, But Not Too Long (2015). Available online at: https://ifstudies.org/blog/want-to-avoid-divorce-wait-to-get-married-but-not-too-long (Accessed October 2, 2025).

[66] Yang T Zhu TS Liu TL. Age at first marriage and divorce risk: analysis based on social clock theory. Fam Relat. (2026) 75:120–38. doi: 10.1111/fare.70096

[67] Bartle-Haring S Shannon S Holowacz E Patton R Lotspeich-Younkin F. Is there the “sweet spot” for age at marriage and positive marital outcomes? J Fam Issues. (2017) 39:1085–107. doi: 10.1177/0192513X16686135

[68] Wooldridge JM. Econometric Analysis of Cross Section and Panel Data (2nd ed.). Cambridge, MA: MIT Press (2010).

[69] Zhang YF Jiang CM Cai R. Study on characteristics of the urban and rural Chinese nationals' physical exercise level. J Tianjin Univ Sport. (2012) 27:306–10, 54. doi: 10.13297/j.cnki.issn1005-0000.2012.04.010

[70] Li XY Zhang XJ. The impact of income and agricultural production categories on nutrition intake of rural residents in China. J Huazhong Agric Univ. (2020) 4:37–49, 175–76. doi: 10.13300/j.cnki.hnwkxb.2020.04.005

[71] Zhao R Zhao L Gao X Yang F Yang Y Fang H . Geographic variations in dietary patterns and their associations with overweight/obesity and hypertension in China: findings from China nutrition and health surveillance (2015–2017). Nutrients. (2022) 14:3949. doi: 10.3390/nu14193949 36235601 PMC9572670

[72] Wang L Zhou B Zhao Z Yang L Zhang M Jiang Y . Body-mass index and obesity in urban and rural China: findings from consecutive nationally representative surveys during 2004–18. Lancet. (2021) 398:53–63. doi: 10.1016/S0140-6736(21)00798-4 34217401 PMC7617101

[73] Wickrama KAS Lorenz FO Conger RD Elder GHJr. Marital quality and physical illness: a latent growth curve analysis. J Marriage Fam. (1997) 59:143–55.

[74] Kittel F Rustin RM Dramaix M de Backer G Kornitzer M. Psycho-socio-biological correlates of moderate overweight in an industrial population. J Psychosom Res. (1978) 22:145–58. doi: 10.1016/0022-3999(78)90018-1 712650

[75] Brown A Siahpush M. Risk factors for overweight and obesity: results from the 2001 national health survey. Public Health. (2007) 121:603–13. doi: 10.1016/j.puhe.2007.01.008 17570451

[76] Ballard J Wieling E Solheim C Lang D. The double ABC-X model of family stress. In:Lang D, editor. Parenting and family diversity issues. Iowa State University Digital Press (2020). Available online at: https://iastate.pressbooks.pub/parentingfamilydiversity/chapter/the-double-abcx-model-of-family-stress/

